# Ossifying Fibroma in the Maxilla and Mandible: A Case Report With a Brief Literature Review

**DOI:** 10.7759/cureus.34257

**Published:** 2023-01-27

**Authors:** Midion Chidzonga, Ephraim Sunhwa, Rudo Makunike-Mutasa

**Affiliations:** 1 Oral and Maxillofacial Surgery, University of Zimbabwe Oral Health Centre, Harare, ZWE; 2 Oral and Maxillofacial Surgery, University of Zimbabwe, Harare, ZWE; 3 Histopathology Unit, Department of Laboratory Diagnostics and Investigative Sciences, Faculty of Medicine and Health Sciences, University of Zimbabwe, Harare, ZWE

**Keywords:** benign neoplasm, multiple ossifying fibroma, cemento-ossifying fibroma, fibrous dysplasia, maxilla, mandible, ossifying fibroma, fibro-osseous lesions

## Abstract

Fibro-osseous lesions in the jaw bones include fibrous dysplasia, ossifying fibroma (OF), cemento-ossifying fibroma, florid osseous dysplasia, and focal osseous dysplasia. OF is the most common fibro-osseous tumor that presents as a slow-growing well-encapsulated benign neoplasm composed of varying amounts of bone or cement-like tissue in a fibrous stroma well-demarcated from the adjacent normal bone. OF is most common in the jaw bones, with a predilection for the mandible. OF usually occur as solitary lesions and rarely as multiple lesions in a patient. We present clinical and radiologic features, histopathology, and surgical management of a rare case with large synchronous OFs in the mandible and maxilla and a brief review of the literature.

## Introduction

Fibro-osseous lesions (FOL) of the jaw bones include fibrous dysplasia, ossifying fibroma (OF), florid osseous dysplasia, cemento-ossifying fibroma (COF), and focal osseous dysplasia [1].

OF is the most common fibro-osseous tumor that presents as a slow-growing, encapsulated benign neoplasm composed of varying amounts of bone or cementum-like tissue in a fibrous stroma well-demarcated from adjacent normal bone [2]. It is most common in the jaw bones, with a predilection for the mandible. Due to the presence of cementum-like tissue and bone in OF, the term OF or COF is sometimes used to describe this tumor. However, the consensus is that these terms describe similar underlying histology and are thus the same underlying type of lesion [2]. The cell of origin is unknown, although it is presumed to arise from multipotential mesenchymal cells in the periodontal ligament space [3]. OF occurs as solitary lesions and rarely as multiple lesions [1]. However, Makkadi et al. [1] reported a rare case with multiple FOL of the jaws in four quadrants and quoted other authors who have reported cases of OF in multiple sites: Takeda and Fujioka [4] in three quadrants; Hauser et al. [5], one case of bilateral lesions in the maxillary sinuses; Hwang et al. [6], one case in all four quadrants; Bertolini et al. [7], one in the maxilla and mandible; Canger et al. [8], two cases of multiple familial OFs involving two quadrants in the mandible and maxilla.

OF may grow to massive sizes resulting in aesthetic and functional deformities. We present the clinical and radiologic features, histopathology, and surgical management of a rare case with massive synchronous OF in the mandible and maxilla and a brief review of the literature.

The Joint Research Ethics Committee of the University of Zimbabwe Faculty of Medicine and Health Sciences and the Parirenyatw Group of Hospitals approved the publication of this case. The patient consented to the publication of her case, including the anonymized photographs.

## Case presentation

On February 14, 2022, a 54-year-old female attended the University of Zimbabwe Oral and Maxillofacial Surgery Clinic, complaining of a slow-growing mass on the mandible and left maxilla for the past three years. Her major complaints were increasing facial disfigurement, pain, difficulty in mouth opening, nasal stuffiness, and problems in breathing and feeding. Her general medical and family history was non-contributory. However, her surgical history was that she had mandibular surgery in 2008, 2012, and 2014 during which parts of her mandible were removed for a tumor. She recalls the cancer was called "ameloblastoma." Unfortunately, no records are available to substantiate this history.

**Figure 1:**
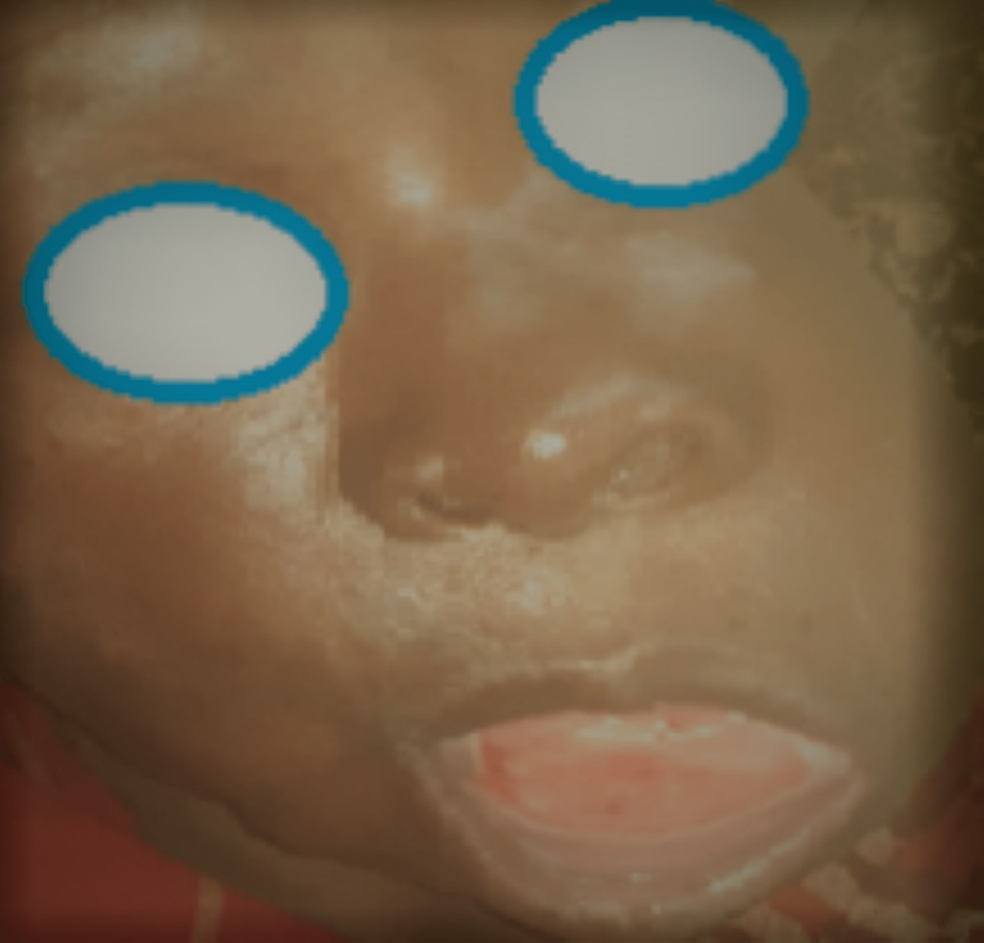
View showing facial disfigurement from maxillary OF. OF: ossifying fibroma

Extra oral examination showed facial disfigurement/asymmetry, limited mouth opening, mild left eye proptosis, and firm bony hard, non-fluctuant, and non-tender tumors in the maxilla and mandible. Intraoral examination showed the tumor extending from the right mandibular second molar to the angle of the left mandible filling both the lingual and buccal sulci. In the maxilla, the non-tender, bony hard tumor extended from the right maxillary canine, crossing the palatal midline and filling the left maxillary buccal sulcus to the tuberosity and extending upwards to the left lateral nasal area and infraorbital margin, extending medially to the zygomatic arch (Figure 1).

The adjacent dentition in the mandible and maxilla was deranged along with distortion of alveoli but with normal oral mucosa covering. Provisional differential diagnoses were of FOL: fibrous dysplasia, OF, central giant cell granuloma, and benign odontogenic tumor.

Orthopantomography (OPG) of the mandible and maxilla showed large well-encapsulated tumors with well-demarcated sclerotic border, displaced teeth, and mixed radiolucent and radiopaque lesions with a peripheral radiolucent area.

**Figure 2:**
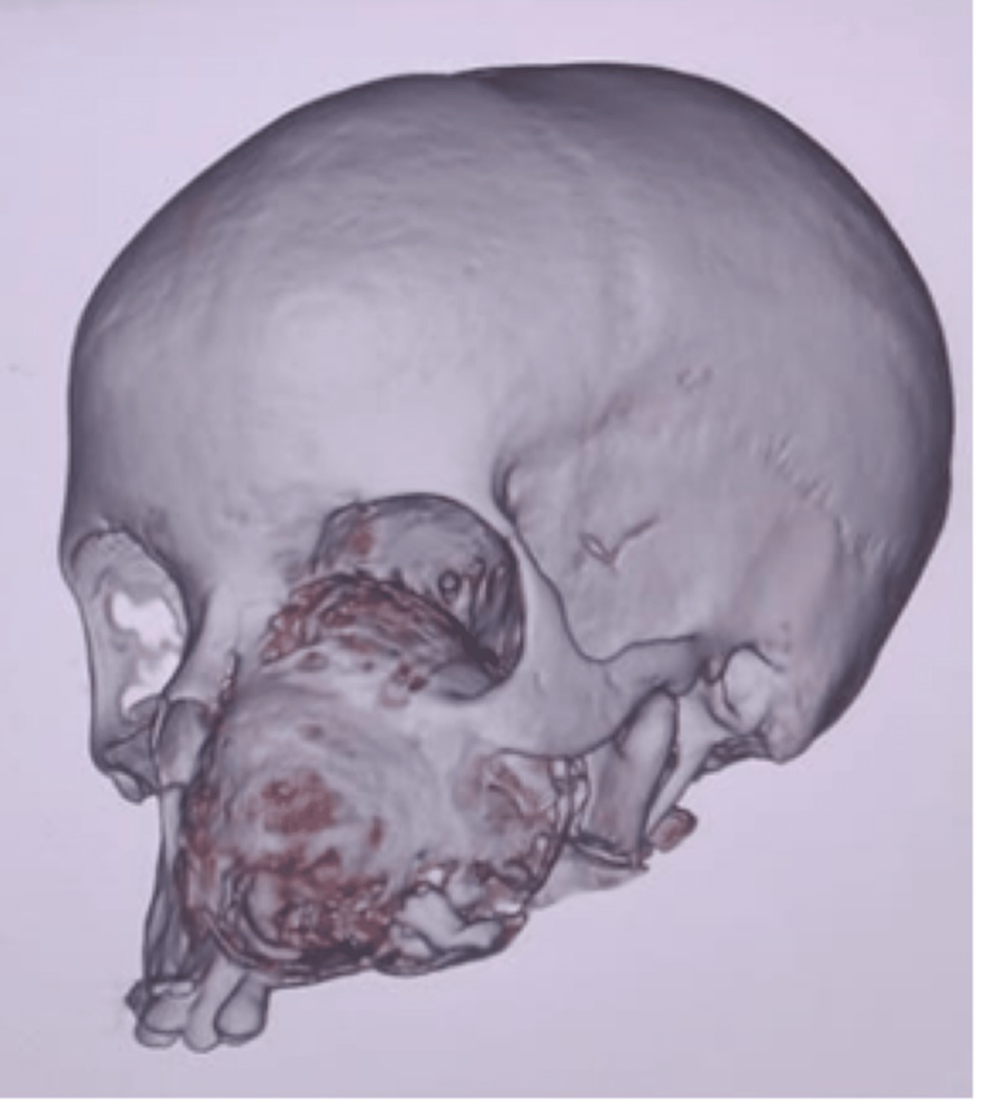
CT scan showing OF in the maxilla. The mandibular OF had been resected during the first surgery. CT: computed tomography; OF: ossifying fibroma

**Figure 3:**
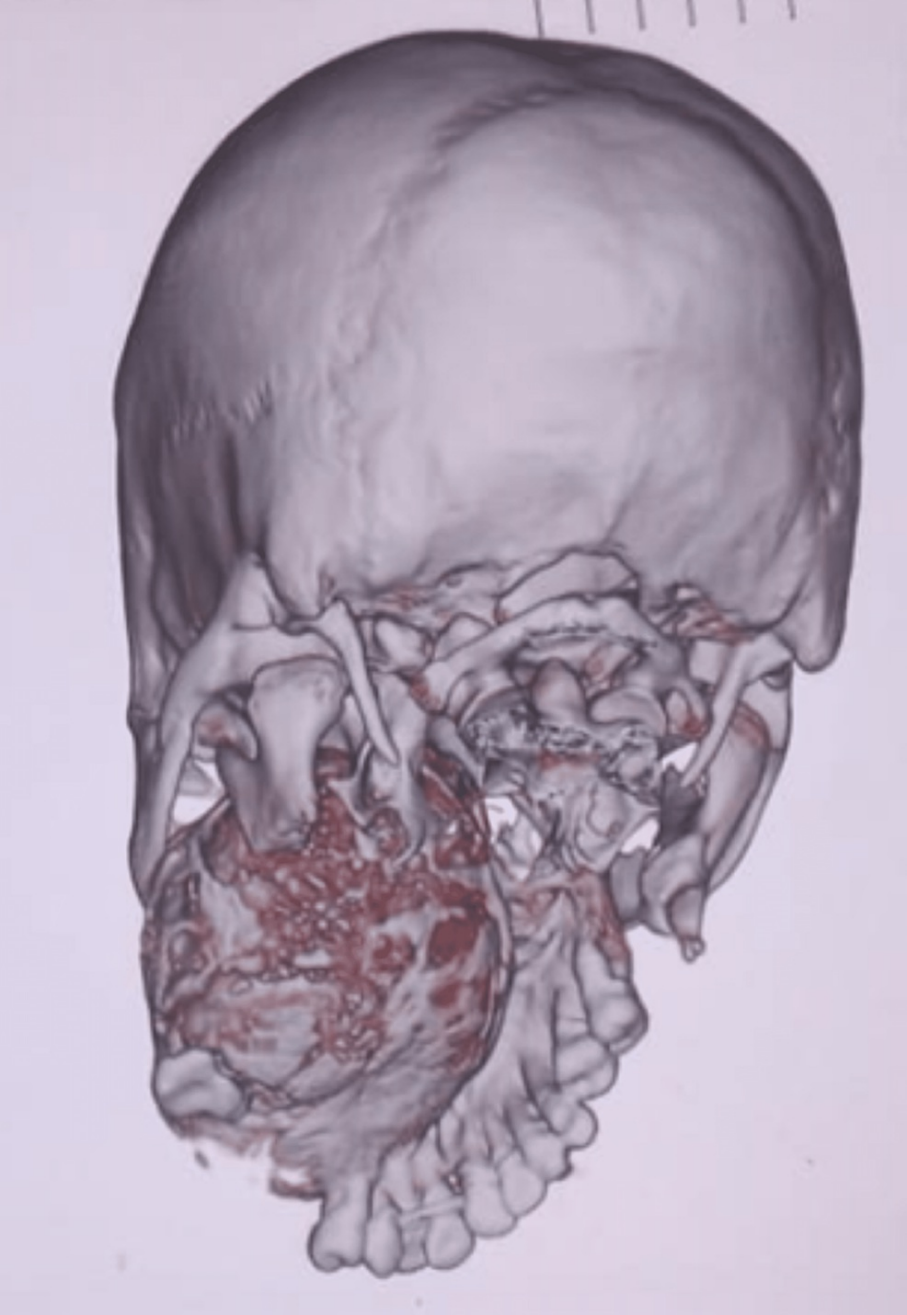
CT scan showing OF in the maxilla. The mandibular OF had been resected during the first surgery. CT: computed tomography; OF: ossifying fibroma

The mandibular computed tomography (CT) scan could not be located at the time of writing this article. The CT scans of the maxillary tumor are shown in Figure 2 and Figure 3.

In the unenhanced CT studies, axial images showed an expansile intramedullary left maxilla tooth-bearing alveolar process tumor with both soft tissue components and snowflake-like calcification within the core of the tumor. At its most expansive dimensions, the tumor averages 8.5 x 6.6 x 5.6 cm in the sagittal, axial, and anteroposterior dimensions. The tumor has expanded into the oral cavity, the maxillary sinus, and the left ethmoidal sinus and obliteration of the hard palate. The left nasal cavity has also been obliterated, and the expansion goes into the left nasopharynx and superiorly bulges into the left orbit with resultant left-sided proptosis. The nasal septum has deviated to the proper alveolar process and has been destroyed by the tumor. The left sphenopalatine fossa has been obliterated, and the tumor expands into the left pterygoid fossa and abuts the left mandible condyle. Antero-superiorly it bulges into the left orbit with some degree of proptosis. The temporomandibular joint is preserved. No aggressive or distinct suprahyoid neck lymphadenopathy, vascular masses, or areas of bleeding are detected to raise malignancy concerns. The petrous mastoid temporal bones bilaterally are preserved. No evidence of intracranial spread of the tumor is noted. The suprahyoid neck and brain are typical. The mandible is missing due to the initial surgery for the mandible.

Consent for tracheostomy and surgery was obtained from the patient. Due to the enormous size of the tumors, phased resection was adopted, starting with the resection of the mandibular tumor. Unfortunately, the mandibular and maxillary tumors filled the oral cavity, and the maxillary tumor compressed and closed the nasal apertures making it impossible to pass a naso-endotracheal or oral tube for intubation. Therefore, a tracheostomy was fashioned to facilitate endotracheal intubation to administer general anesthesia and maintain the airway.

Resection en bloc under general anesthesia was indicated due to the size of the tumor and the fact that the tumor had breached the upper and lower borders of the mandible with simultaneous expansion of the mandible. The tumor was approached through a lower lip midline split incision along with right and left submandibular incisions. Blunt dissection was used to shell out the tumor with mandibular resection at the right and left angles of the mandible. The excised mandibular tumor had a K-nail in situ. The K-nail is presumably from previous mandibular resection. This presumably is a recurrent OF based on the patient's surgical history. Due to the unavailability of reconstruction plates, a K-nail was used to stabilize the remnants of the mandible. Immediate bone reconstruction was contraindicated due to possible infection and the fact that the maxillary OF still had to be resected en block. The patient tolerated the procedure well.

Macroscopic examination showed a specimen of a hemi-mandible measuring 101 x 54 x 50 mm and a tab grey hard tumor measuring 78 x 75 x 88 mm. The cut surface of the tumor was a homogenous grey. Microscopic examination showed abundant fibrous stroma with scant rounded spicules of mature bone deposits consistent with OF.

**Figure 4:**
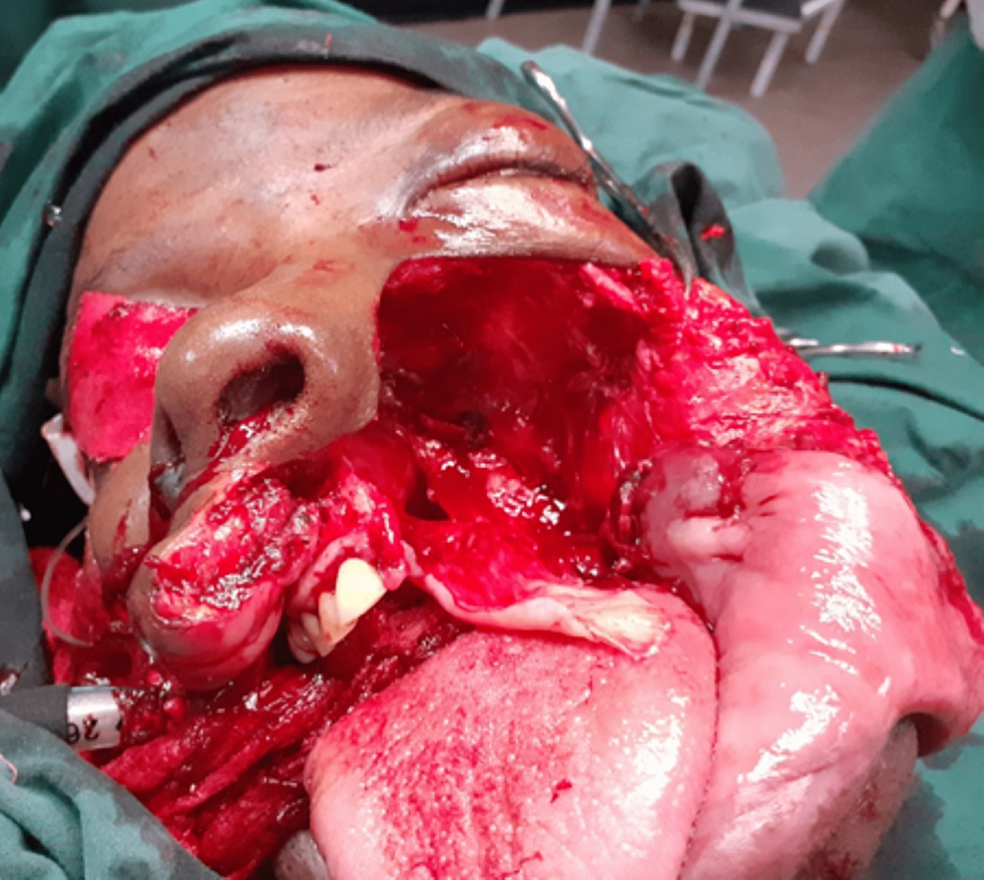
Intraoperative view of the bed of the maxillary OF. OF: ossifying fibroma

Four weeks post mandibular resection, the maxillary tumor was resected under oral endotracheal intubation general anesthesia, as intraoral access was now possible after the mandibular resection. Maxillary resection was achieved through the extended Weber-Ferguson incision. Figure 4 shows the intraoperative bed of the tumor.

**Figure 5:**
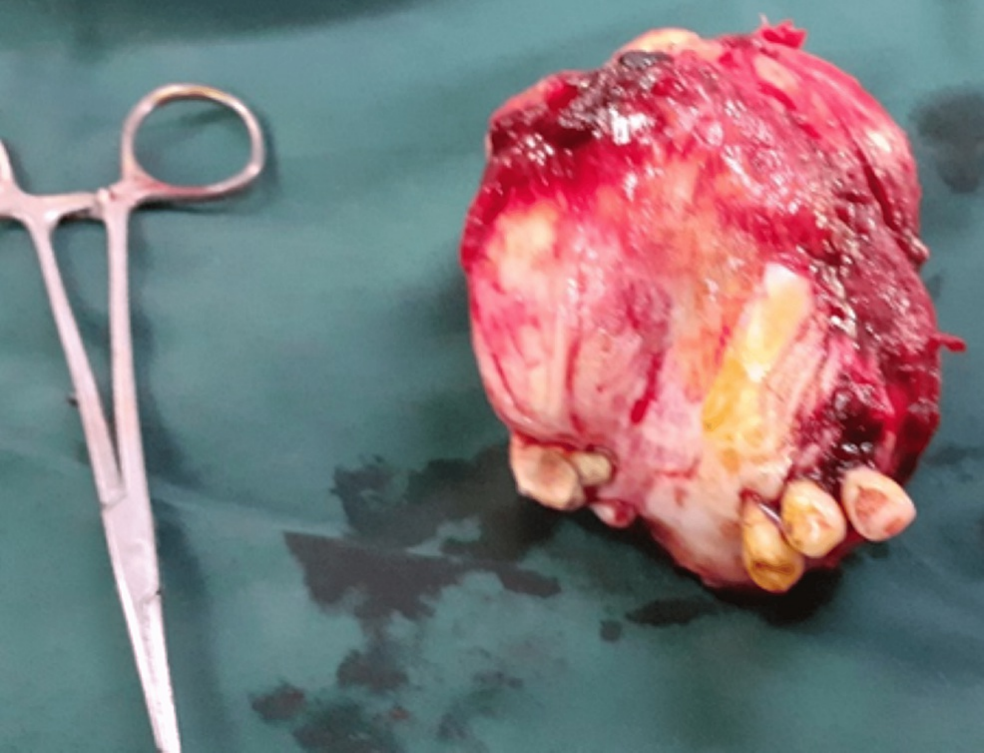
Excised maxillary OF. OF: ossifying fibroma

The tumor covered three-quarters of the palate and was resected en-block (Figure 5).

The tumor had extended into the left maxillary sinus and the left nasal cavity to the infraorbital margin, the floor of the orbit, and behind the lateral wall of the left orbit. The mucosa overlying the tumor was preserved by shelling it away from the tumor with a blood supply base from the remaining quarter of the palate. The covering mucosa was used to line up the bony defect planned for an obturator or bone grafting at a later stage. However, immediate reconstruction with bone was deferred to avoid possible infection and recurrence, and the thinned mucosa may dehisce over the bone graft. Figure 4 shows the intraoperative view post tumor resection. The defect was packed with povidone iodine-soaked paraffin gauze. The maxillary tumor was a well-encapsulated firm grey tumor on the part of the maxilla measuring 80 x 75 x 60 mm (Figure 5).

The cut surface of the tumor was a homogenous grey-white with a gritty sensation when cutting.

**Figure 6:**
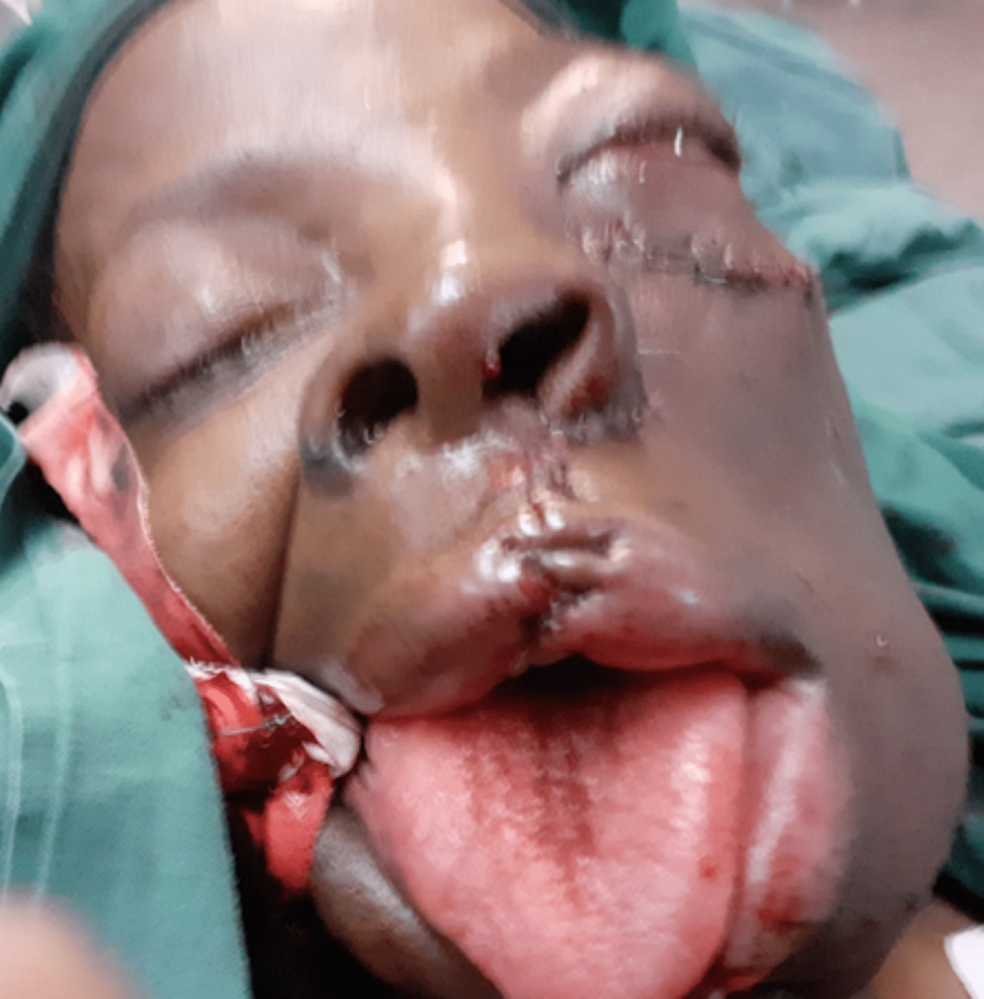
Immediate postoperative view after partial maxillectomy of the maxillary OF. OF: ossifying fibroma

Figure 6 shows the immediate postoperative view of the patient.

**Figure 7:**
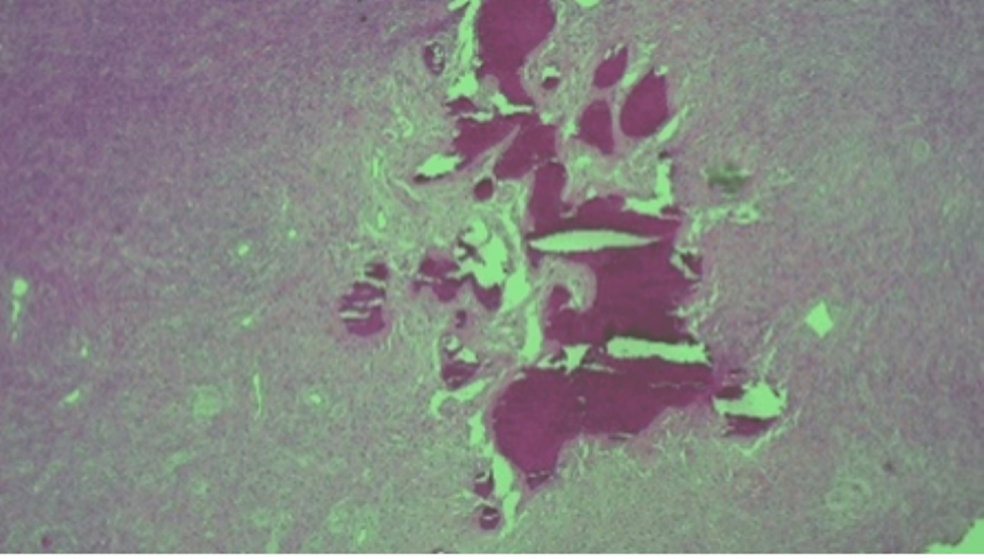
Histopathologic photomicrograph of the maxillary OF at 4X magnification, hematoxylin stain. OF: ossifying fibroma

**Figure 8:**
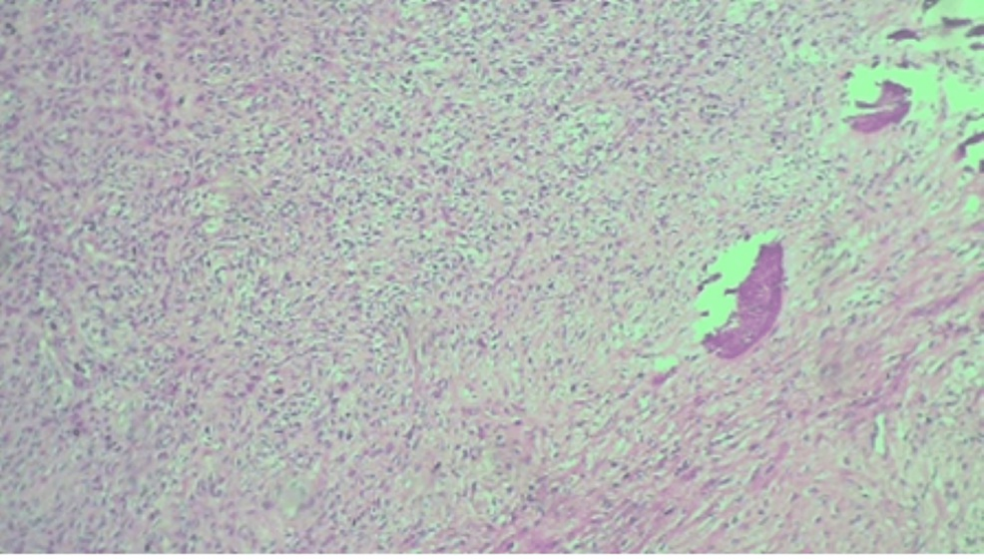
Histopathologic photomicrograph of the specimen at 10X magnification with hematoxylin stain.

**Figure 9:**
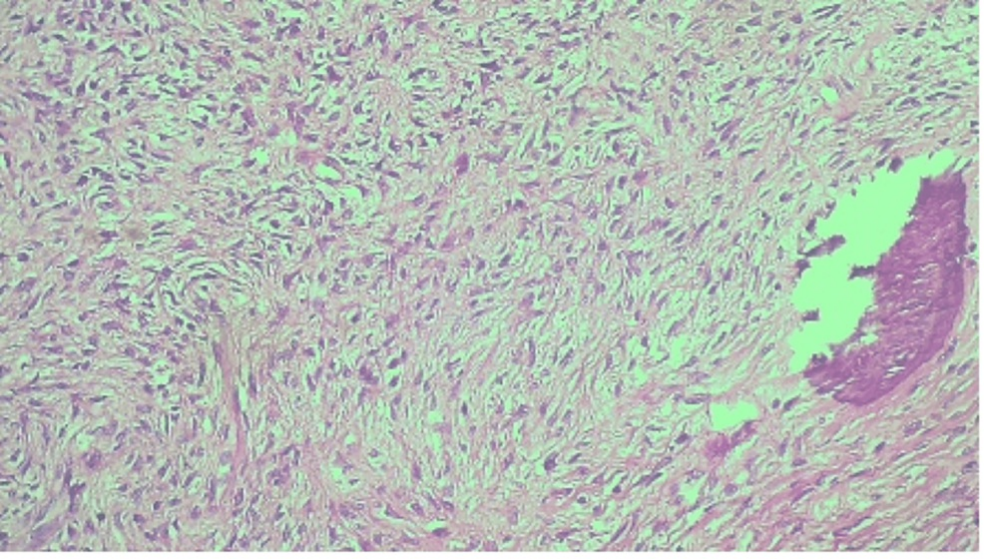
Histopathologic photomicrograph of the maxillary OF at 20X magnification with hematoxylin-eosin stain. OF: ossifying fibroma

Microscopically, sections showed a well-circumscribed tumor comprising bone and collagen components. There are bone spicules with a surrounding fibrous cellular stroma consisting of spindle cells with abundant collagen. There was no nuclear pleomorphism, mitosis, or necrosis. Figures 7, 8, 9 are the photomicrographs of the hematoxylin stain of the maxillary OF.

The clinical, radiographic, and histologic features are of a FOL consistent with OF, a multiple conventional OF in both the mandible and maxilla.

**Figure 10:**
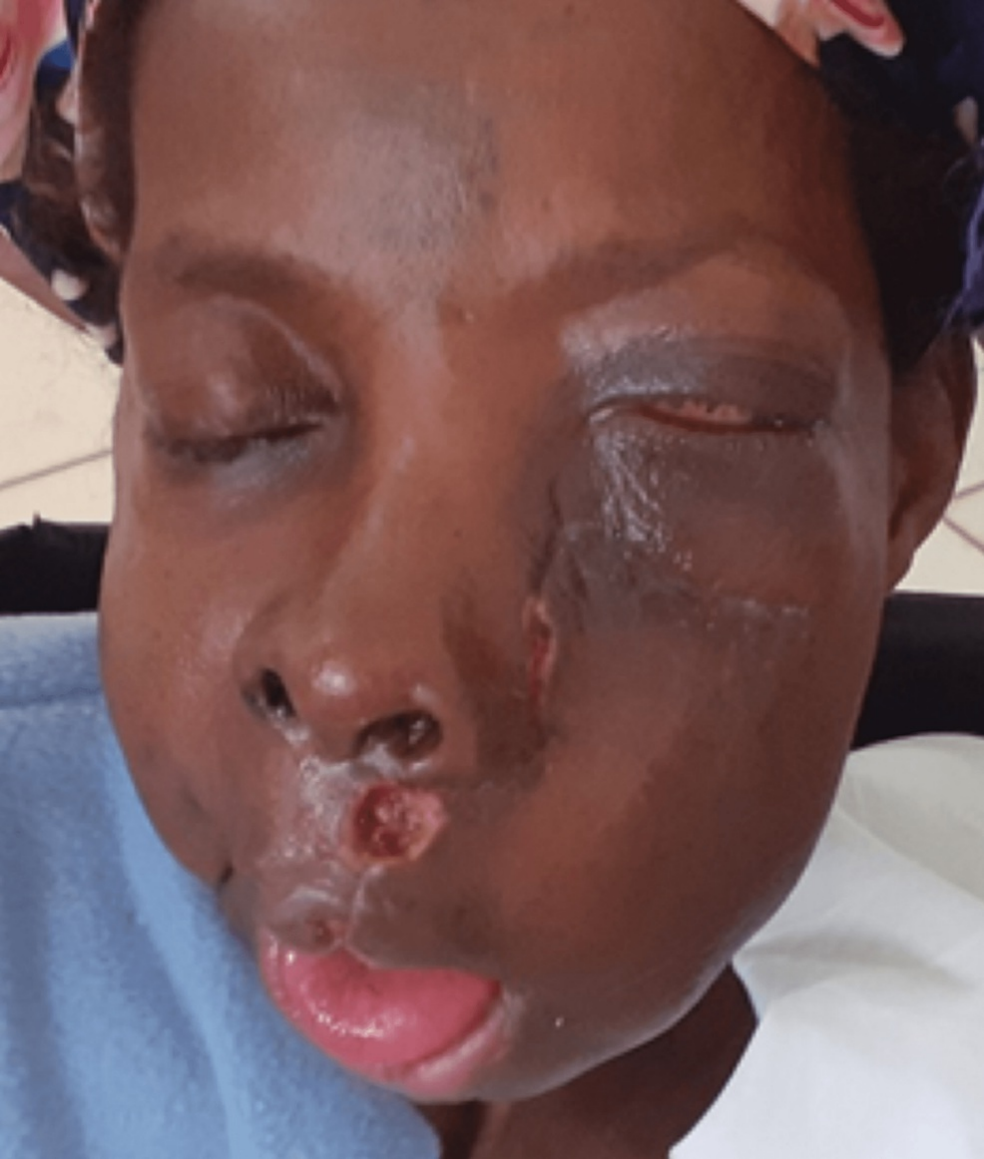
View of the patient three weeks postoperatively.

Figure 10 shows the view of the patient three weeks postoperatively.

## Discussion

FOL are a rare group of lesions of unclear etiohistogenesis. OF is a benign bone tumor, most frequent in children and most common in the maxillary sinus and the mandible (75-89%). Sciubba et al. [9] and Yih et al. [10] attributed the first case of OF to Menzel in 1872 as a rare benign primary craniofacial skeleton bone tumor that most commonly affects the jaws [11]. The term OF was first used in 1927 by Montgomery [12]. The 4th Edition of the World Health Organization Classification of Head and Neck tumors described odontogenic and maxillofacial bone tumors classify OF under fibro and chondro-osseous lesions along with familial gigantiform cementoma, fibrous dysplasia, cemento-osseous dysplasia and osteochondroma [13]. Three variants of OF are known: COF, juvenile trabecular OF (JTOF), and juvenile psmammomatoid OF (JPOF). COF has an odontogenic origin and is slow-growing with a predilection for the posterior mandible and rarely the maxilla with female predilection [14]. JPOF is rare, with a mean age range of 16-33 years, and occurs mainly in the extragnathic regions of the craniofacial bones with predominance in the orbit and ethmoids [15]. JPOF, more aggressive, presents with a well-delineated periphery with mixed radiopacity and radiolucency. The treatment of JTOF and JPOF is complete resection, although recurrence is higher than in COF. Seventy percent of OF involve the mandible, with 22% found in the molar region of the maxilla, the ethmoids, orbital regions, and rarely the petrous bone [1]. OF commonly occurs as a solitary lesion, although rarely seen in multiple sites or shows multiple, familial, or sequential presentations [1].

Pathogenesis of OF has not been concretized, with several theories being advanced. OF develops from pluripotential mesenchymal cells capable of forming cementum, bone, and fibrous tissue [11]. Two possible origins have been suggested: an excessive proliferation of the periodontal ligament cells and a metastatic process occurring in the connective tissue fibers (non-periodontal) in origin [11]. The jaws are commonly affected, possibly due to the extensive mesenchymal cellular induction into bone and cementum during odontogenesis [11]. Trauma, previous extraction, periodontitis, and possible genetic defects are being investigated, especially mutations of the hyperparathyroidism 2 (HRPT2) gene have been suggested as potential causative agents. OF occurs at any age but usually in children and young adults with a predilection for the mandible and rarely the maxilla, with a female predominance in the third and fourth decades of life [14,5]. It has also been reported in the paranasal sinuses, frontal, ethmoid, sphenoid bone, and the orbital roof. Our case is a 54-year-old female with massive OF in the maxilla and mandible. Clinically OF presents as an asymptomatic slow, growing, occasionally aggressive, particularly in its juvenile subtypes, or accidentally on routine dental examinations [2,3]. Growth is usually concentrically arising apically to the premolars and molars superior to the mandibular canal. The overlying mucosa or skin and the cortical bone plates of the bone are invariably intact [16,17]. In our case, the extensive tumors in multiple sites had grown over a long period with a history of surgical interventions. The clinicopathologic characteristics of multiple OF are unclear due to the condition’s rarity, making diagnosis challenging [18]. The spectrum of differential diagnoses includes benign lesions: osteomas and osteoid osteomas, reactive expansile non-neoplastic lesions, primary malignant tumors, and metastatic lesions [19]. FOL are rare. However, sporadic multiple cases OFs must be distinguished from hyperparathyroidism-jaw tumor syndrome (HPT-JT) related to OF and other FOL [18].

CT scans and magnetic resonance imaging (MRI) help contribute to the final diagnosis of OF. The radiographic features of OF are usually a round or oval, relatively smooth, well-defined, expansile mass with a corticated border and variable degree of internal radiopacity [2,3]. The inner aspect can be granular, as in fibrous dysplasia, or have a radiolucent periphery representing a fibrous capsule, cortical bone expansion, and a mixture of radiolucent and radiopaque tissue. The mandibular canal near the tumor is usually inferiorly displaced. OPG shows a well-defined unilocular mixed lesion with a ground-glass appearance internally surrounded by a thin radiopaque margin; root resorption may be present [19,20]. In our case, the OPG showed large well-encapsulated, well-demarcated, mixed radiolucent and radiopaque lesions with a peripheral radiolucent area in the maxilla and mandible. A coronal cone-beam computed tomography (CBCT) may show a concentric corticated lesion with radiopaque foci with an expansion of the involved bone. A sagittal CBCT will reveal a well-defined radiolucent lesion, and axial CBCT also shows an expansion of bone [19]. Digital volumetric tomography (DVT) shows expansion and thinning of intact cortical bone with radiopaque calcified within an internal structure in the axial view, with a 3D Recon view showing mixed density due to a variable amount of radiopaque material. On CT scan, OF usually appears as an expansile well-circumscribed lesion with a typical aspect of ground glass appearance that could contain a cystic component [15]. In our case, CT scan axial and coronal images showed an expansile osteogenic lesion in the maxilla (left maxilla lesion extending upwards to the infraorbital margin and lateral to the left orbit and left lateral nasal wall) and in the mandible (extending from distal to the third molar to the left temporomandibular joint and showing well-delineated spherical margins and a heterogenous density). MRI shows intensities similar to the brain gray matter on precontrast T1-weighted images and intermediate to low intensities on T2-weighted sequences; after gadolinium injection, there is an enhancement of the lesion [15].

Histologically, OF presents as a relatively avascular fibrous stroma consisting of fusiform cells intermingled with bone trabeculae and spheroidal calcifications that resemble cement structures [3]. Multinucleated giant cells may be present. The calcified material consists of irregularly shaped trabeculae of woven bone; scattered trabeculae of lamellar bone; deposits of basophilic staining round or oval, cellular, or acellular calcified deposits that have been likened to cementum or any combination [3]. Microscopically, OF has a cellular connective tissue with mineralized material with osteoblastic rimming observed on the surface of the mineralized tissue. This is comparable to the histology and microscopy of our case.

Complete surgical removal with curettage, surgical excision, or en-block resection, depending on the lesion size [3]. Radical surgery is indicated to reduce the tendency of recurrence and the possibility of malignant transformation. A fibrous capsule allows for easy surgical excision and resection, as in our case with the massive tumors - a study of 25 OF patients by Liu et al. [16,20]. The following methods were used: 36% (n=09) in the mandible had enucleation and curettage; 32% (n=03) had segmental mandibular resection and reconstruction with reconstruction plate; with 20% (n=05) reconstructed with microvascular fibula graft. In the maxilla, 20% (n=05) had enucleation and curettage, and 12% (n=03) had partial maxillectomy. In our case, three-quarters of the maxilla had to be resected due to the extent of the tumor. Partial or incomplete resection leads to recurrence. Possibly the “recurrence” mentioned in our case was due to inadequate enucleation. The recurrence period is unpredictable but ranges from six months to seven years after surgery. Therefore, it is recommended to have a follow-up period of up to 10 years.

## Conclusions

Accurate diagnosis may be arrived at by clinical-radiological and histopathological examinations. The synchronous OF of the maxilla and mandible occurring in multiple sites is rare. Radical surgical resection is indicated for extensive lesions while surgical excision is adequate in most OF cases. In order to reduce the risk of recurrence extensive en bloc resection is indicated for extensive lesions.
